# Infliximab-Induced Hypothyroidism: A Novel Case and Postulations concerning the Mechanism

**DOI:** 10.1155/2013/216939

**Published:** 2013-11-19

**Authors:** Brett Cerniglia, Marc A. Judson

**Affiliations:** ^1^Department of Medicine, Albany Medical College, Albany, NY 12208, USA; ^2^Division of Pulmonary and Critical Care Medicine, Albany Medical College, Albany, NY 12208, USA

## Abstract

We report a patient with cutaneous sarcoidosis who developed hypothyroidism following 17 months of infliximab therapy. To our knowledge, this is the first reported case of hypothyroidism following infliximab administration. While it is possible that the patient's hypothyroidism was unrelated to the use of infliximab, the time course and lack of alternative explanations make such an association plausible. We postulate that hypothyroidism in this patient may have been related to the development of autoantibodies to infliximab that triggered the development of an autoimmune thyroiditis. Regardless of the mechanism, we would encourage clinicians to keep the potential mechanisms of TNF-*α* in mind when treating patients with TNF-*α* antagonist medications.

## 1. Introduction

Infliximab is a tumor necrosis factor alpha (TNF-*α*) inhibitor that has been shown to have efficacy for sarcoidosis [1–3]. Potential adverse responses to infliximab include the production of anti-infliximab antibodies [4] and autoantibodies [5] and even the induction of autoimmune diseases [6]. With the established presence of TSH regulated TNF-*α* receptors in human thyroid tissue [7], it is plausible that these adverse immune responses may affect thyroid function. We report a patient with cutaneous sarcoidosis who developed hypothyroidism of unknown cause following continued administration of infliximab.

## 2. Case Report

A 47-year-old white man with biopsy-confirmed sarcoidosis of the nasal sinuses was referred for skin lesions consistent with lupus pernio (disfiguring facial sarcoidosis). The lesions had worsened over the previous five years and were associated with significant nasal congestion. The lesions were initially treated with a six-month course of oral corticosteroids; however, the patient discontinued corticosteroids because of concern for potential side effects. Additionally, the lesions failed to improve with several months of mycophenolate therapy, 500 mg twice daily. His physical examination at presentation was remarkable for indurated violaceous to erythematous lupus pernio lesions on his nose.

The patient was treated with infliximab infusions 5 mg/kg at weeks 0, 2, and 6 and then every 6 weeks for his lupus pernio lesions. By the third infusion, he noticed a remarkable regression in both the lesions and associated nasal congestion. The frequency of his infliximab infusions was lengthened to once every eight weeks and his skin lesions completely resolved.

Seventeen months after initiating infliximab therapy, the patient developed muscle cramping, cold intolerance, a deeper voice, constipation, and nasal congestion. His lupus pernio lesions remained resolved. His resting heart rate was 58/minute and laboratory data was remarkable for a serum creatinine kinase of 410 IU/L, TSH of 137 mIU/L, and free T4 of 0.28 ng/L. An ultrasound of his thyroid showed mild areas of heterogeneity consistent with a chronic autoimmune thyroiditis. However, serum thyroid peroxidase and thyroglobulin antibodies were negative. He was placed on levothyroxine at 125 mcg/day. All his symptoms of hypothyroidism slowly resolved over a few months. Three months after starting levothyroxine therapy, his serum TSH level had fallen to 1.01 mIU/L. His infliximab infusions have been continued.

## 3. Discussion

We report a patient with multidrug resistant lupus pernio who developed a de novo condition of hypothyroidism following the administration of infliximab. While it is possible that the condition developed independently of infliximab administration, the negative blood work and timeline of occurrence are inconsistent with an isolated case of chronic lymphocytic (Hashimoto's) thyroiditis or a sarcoid related granulomatous thyroid involvement, respectively. 

TNF-*α* has been shown to have varied effects on thyroid gland function. These include the presence of TNF-*α* receptors in human thyroid tissue [7], TNF-*α* induced inhibition of thyroid function in rats [8], elevated TNF-*α* and its receptor in cases of hypothyroidism [9], a relationship between TNF-*α* and exacerbations of pre-existing hypothyroidism [10], and the fact that infliximab decreases thyroid hormone replacement requirements in pre-existing hypothyroidism [11]. 

Infliximab is a chimeric antibody with a human and murine portion. Antibodies may develop into infliximab. Although the development of these antibodies may cause no significant medical problems, their formation may lead to adverse drug reactions, treatment failure [4], and the development of autoimmune diseases such as cutaneous vasculitis and lupus-like syndromes [6]. These antibodies typically occur after at least 3 to 6 months of infliximab therapy [12]. We postulate that this patient's infliximab treatment may have led to the production of autoantibodies and, as a result, triggered the induction of an autoimmune thyroiditis. The time course of this patient's hypothyroidism and lack of evidence of other causes of hypothyroidism support this postulation. 

Regardless of the mechanism, our report remains the first to suggest the role of infliximab in the induction of a de novo case of hypothyroidism. We are unaware of any studies concerning thyroid function in sarcoidosis patients treated with infliximab or any other TNF-*α* antagonists. We would encourage clinicians to keep the potential effects of TNF-*α* antagonists on thyroid function including this postulated mechanism in mind when treating patients with infliximab and other TNF-*α* antagonists.
